# Immunization with alkyl hydroperoxide reductase subunit C reduces *Fusobacterium nucleatum* load in the intestinal tract

**DOI:** 10.1038/s41598-017-11127-x

**Published:** 2017-09-05

**Authors:** Song-He Guo, Hai-Fang Wang, Zhi-Gang Nian, Yi-Dan Wang, Qiu-Yao Zeng, Ge Zhang

**Affiliations:** 1 Department of Microbial and Biochemical Pharmacy, School of Pharmaceutical Sciences, Sun Yat-sen University, Guangzhou, China; 2 Department of Clinical Laboratory Medicine, Sun Yat-sen University cancer center, Guangzhou, China, Guangzhou, China; 3 Department of School of Life Science, Sun Yat-sen University, Guangzhou, China

## Abstract

*Fusobacterium nucleatum* (Fn) is an important tumour-associated bacterium in colorectal cancer (CRC). The antioxidant protein alkyl hydroperoxide reductase subunit C (AhpC) can induce strong antibacterial immune response during various pathogen infections. Our study aimed to evaluate the efficacy of Fn-AhpC as a candidate vaccine. In this work, by western blot analysis, we showed that Fn-AhpC recombinant protein could be recognized specifically by antibodies present in the sera of CRC patients; using the mouse Fn-infection model, we observed that systemic prophylactic immunization with AhpC/alum conferred significant protection against infection in 77.3% of mice. In addition, we measured the anti-AhpC antibody level in the sera of CRC patients and found that there was no obvious increase of anti-AhpC antibodies in the early-stage CRC group. Furthermore, we treated Fn with the sera from both immunized mice and CRC patients and found that sera with high anti-AhpC antibodies titre could inhibit Fn growth. In conclusion, our findings support the use of AhpC as a potential vaccine candidate against inhabitation or infection of Fn in the intestinal tract, which could provide a practical strategy for the prevention of CRC associated with Fn infection.

## Introduction

The gram-negative anaerobe *Fusobacterium nucleatum* (Fn) is an important tumour-associated bacterium that promotes colorectal tumour growth and inhibits T cell-mediated immune responses against colorectal tumours^1, 2^. A number of studies have identified the increased carriage of Fn in tumour tissues and faecal samples of colorectal cancer (CRC) patients^3, 4^ and demonstrated an association of Fn overabundance with shorter survival rates of CRC patients^5^. Faecal Fn infection has been identified as an important diagnostic marker for CRC^6, 7^. Previously, Fn was considered a key aetiological agent in human periodontal disease^8–10^ that plays important roles in different infectious processes, including juvenile idiopathic arthritis, rheumatoid arthritis and Alzheimer’s disease^11–13^. Recently, accumulating evidence has shown a high correlation between Fn infection and gastrointestinal tumours, and novel strategies on cancer prevention and treatment by targeting Fn have been proposed^2, 14^.

Previous studies have shown that Fn induced a significant humoural immune response in chronic oral infection^12, 15^. In a recent study, we showed that Fn infection elicited high-level serum antibody to Fn in CRC patients^16^. Using the sera from CRC patients to probe the bacteria protein extract, we identified a strong reactive antigen, alkyl hydroperoxide reductase subunit C (AhpC), which triggers the anti-Fn immune response^16^. The AhpC protein is a member of the highly conserved family of peroxiredoxins that catalyse the reduction of hydroperoxides for protection against oxygen radical damage^17^. AhpC is present in most bacterial species, which is particularly important for the role of protecting cells against organic peroxides in obligate anaerobes by eliminating hydroperoxides. AhpC has been identified as a potent immunoantigen that induces strong T cell-mediated responses in patients with acute melioidosis^18^. Recently, the antioxidant protein AhpC was considered a potential vaccine candidate against various bacterial infections, including *Helicobacter pylori, Bacillus anthracis, Streptococcus zooepidemicus, Salmonella typhimurium* and *Burkholderia pseudomallei* ^18–22^.

In the present study, we developed a recombinant AhpC vaccine and examined the protective efficacy against Fn overload in the murine intestinal tract. We further evaluated the prevalence of serum antibodies to Fn-AhpC in CRC patients and investigated the role of anti-Fn-AhpC antibodies against Fn *in vitro*.

## Results

### Identification of AhpC as vaccine candidate antigen for *F. nucleatum*

**Figure 1:**
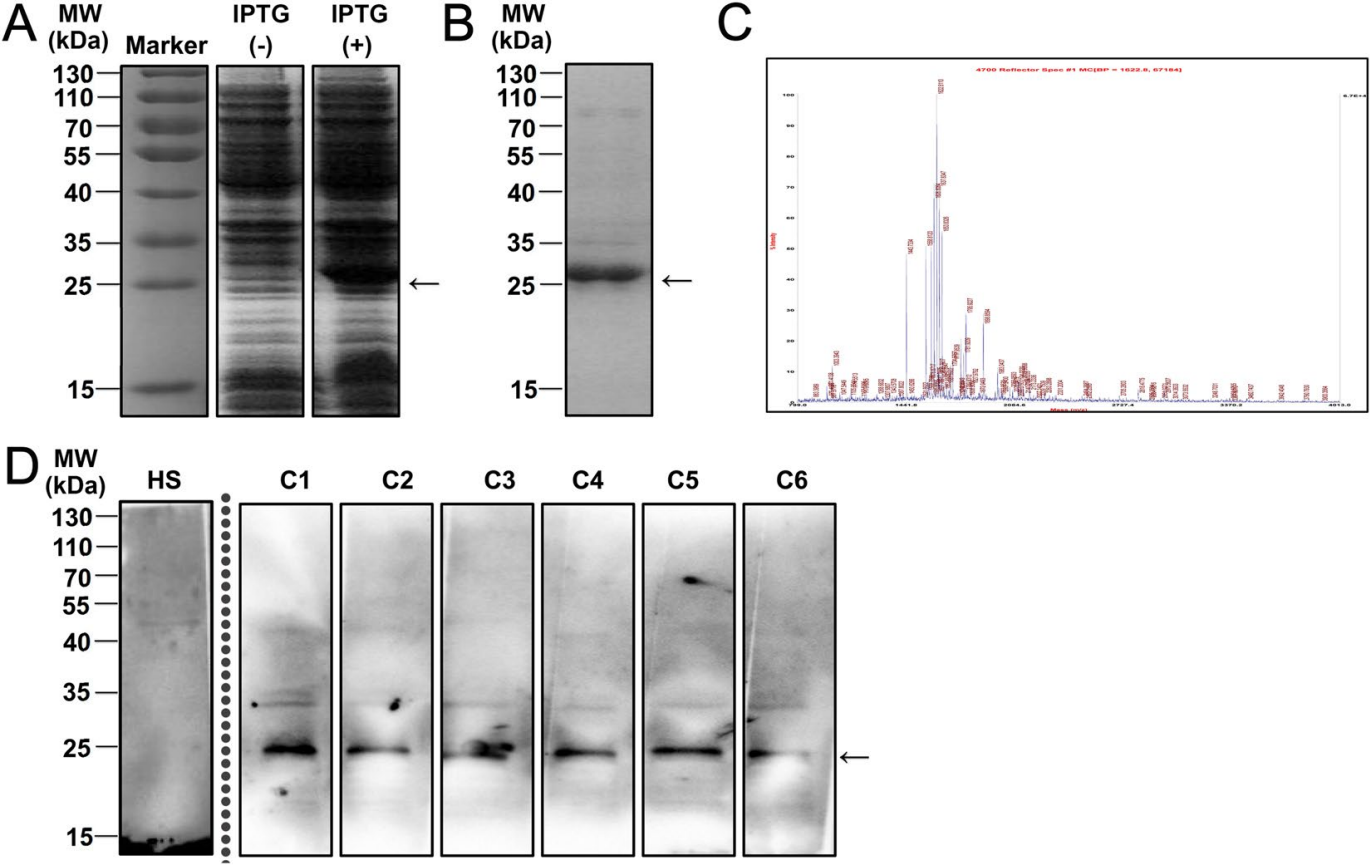
Preparation of recombinant *F. nucleatum*-AhpC and identification of its immunogenic role in CRC patients. (**A**) Expression of recombinant Fn*-*AhpC after IPTG induction. The PCR amplified AhpC-DNA was inserted into pET28a vector before transformed into *E. Coli* BL21 strain. The expression of recombinant AhpC in the absence (−) or presence (+) of 0.5 mM IPTG was detected using 12% SDS-PAGE and Coomassie brilliant blue staining. (**B**) Purification of recombinant AhpC. The recombinant AhpC with a His tag was purified using a Ni-NTA column. (**C**) Identification of recombinant AhpC. The recombinant AhpC proteins were extracted from the gels stained with Coomassie brilliant blue R250, and subsequently digested with trypsin. The resulting peptides were further analysed using a MALDI-TOF/TOF analyser. (**D**) Antigens reactive with anti*-*AhpC-IgA were determined using western blotting. Recombinant AhpC were incubated with a reference dilution of pooled serum from 6 healthy subjects or separated serum from 6 Fn-positive CRC individuals as primary antibody. Notably, Fig. 1A,B and D were cropped from a single image on the dashed or solid lines to be better presented in the article’s context. The complete figures for Fig. 1A,B and D are provided in Supplementary Fig. 1A, Supplementary Fig. 1B and Supplementary Fig. 2, respectively.

To identify anti-Fn vaccine candidates, we measured the pooled sera from 6 Fn-positive CRC patients using western blotting to probe the bacterial protein, as previously described^16^. We observed that AhpC protein induced a much stronger immune response than FomA protein, although a lower level of AhpC was presented in the total protein of Fn compared to FomA^16^. Next, we constructed a prokaryotic expression plasmid pET28a-AhpC to prepare recombinant Fn-AhpC protein. The overexpressed AhpC-His fusion proteins were detected at approximately 25 kDa (Fig. 1A). After IPTG induction, the soluble AhpC was purified using a Ni-NTA agarose column and was validated using a MALDI-TOF/TOF analyser (Fig. 1B,C). The purified Fn-AhpC reacted strongly with the pooled sera from CRC patients, whereas no immunoreactivity was detected in the sera from healthy subjects, indicating the antibodies present in the sera of CRC patients specifically recognized the Fn-AhpC proteins (Fig. 1D).

**Figure 2:**
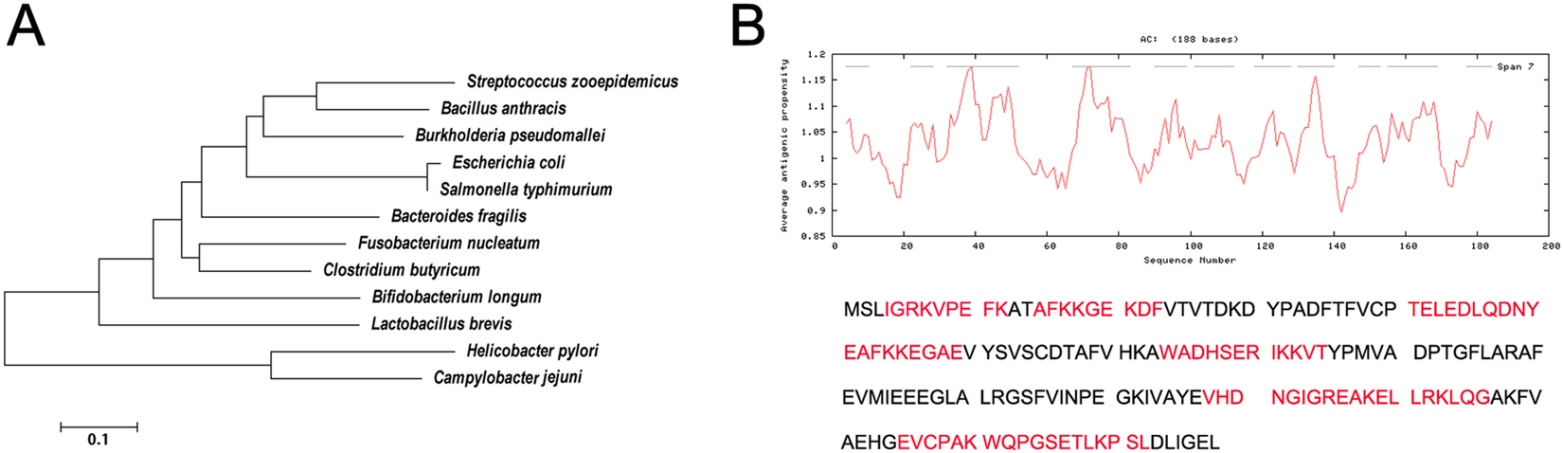
Phylogenetic tree analysis and antigenic determinants prediction for AhpC of *F. nucleatum*. (**A**) Phylogenetic tree of AhpC amino acid sequences. The amino acid sequences of AhpC present in protein database of GenBank were analysed using MEGA version 5.1 (www.megasoftware.net). The numbers at each branch represent the bootstrap values obtained with 1000 replicates. (**B**) The improved self-optimized software (DNAStar Protean system) was used to predict the antigenic plot for Fn-AhpC protein. Average antigenic propensity for this protein is 1.0330.

Furthermore, we constructed a phylogenetic tree based on the alignment of AhpC amino acid sequences, and the results showed an evolutionary relationship between Fn and other species (Fig. 2A). BLASTP analysis revealed that Fn-AhpC exhibited a low level of sequence identity with the human AhpC protein (31%). In addition, Fn-AhpC showed low identity with *H. pylori* (34.6%) and *Campylobacter jejuni* (37.2%), and moderate identity with *S. zooepidemicus* (56%), *B. anthracis* (59%), *S. typhimurium* (59%) and *B. pseudomallei* (61.7%). Antigenic peptide prediction suggested that Fn-AhpC possessed multiple cell antigenic determinants, indicating that Fn-AhpC had the capacity to induce an intense immune response (Fig. 2B).

Interestingly, although Fn-AhpC also shared moderate amino acid identity with intestinal commensal bacteria, such as *Lactobacillus brevis* (53%), *Bifidobacterium longum* (58%), *Escherichia coli* (59%), *Bacteroides fragilis* (61%), and *Clostridium butyricum* (68%) (Fig. 2A), the potential antigenic determinants in AhpC differed among these microorganisms (Supplementary Table 1), suggesting that the AhpC protein of *F. nucleatum* induces few immune cross-reactions with these commensal bacteria (Fig. 2B). However, further studies are required to empirically demonstrate this finding. The results also showed that Fn-AhpC is a potential candidate antigen to develop the vaccine or diagnostic reagent against Fn.

### Protective efficacy of AhpC antigen against *F. nucleatum* challenge

**Figure 3:**
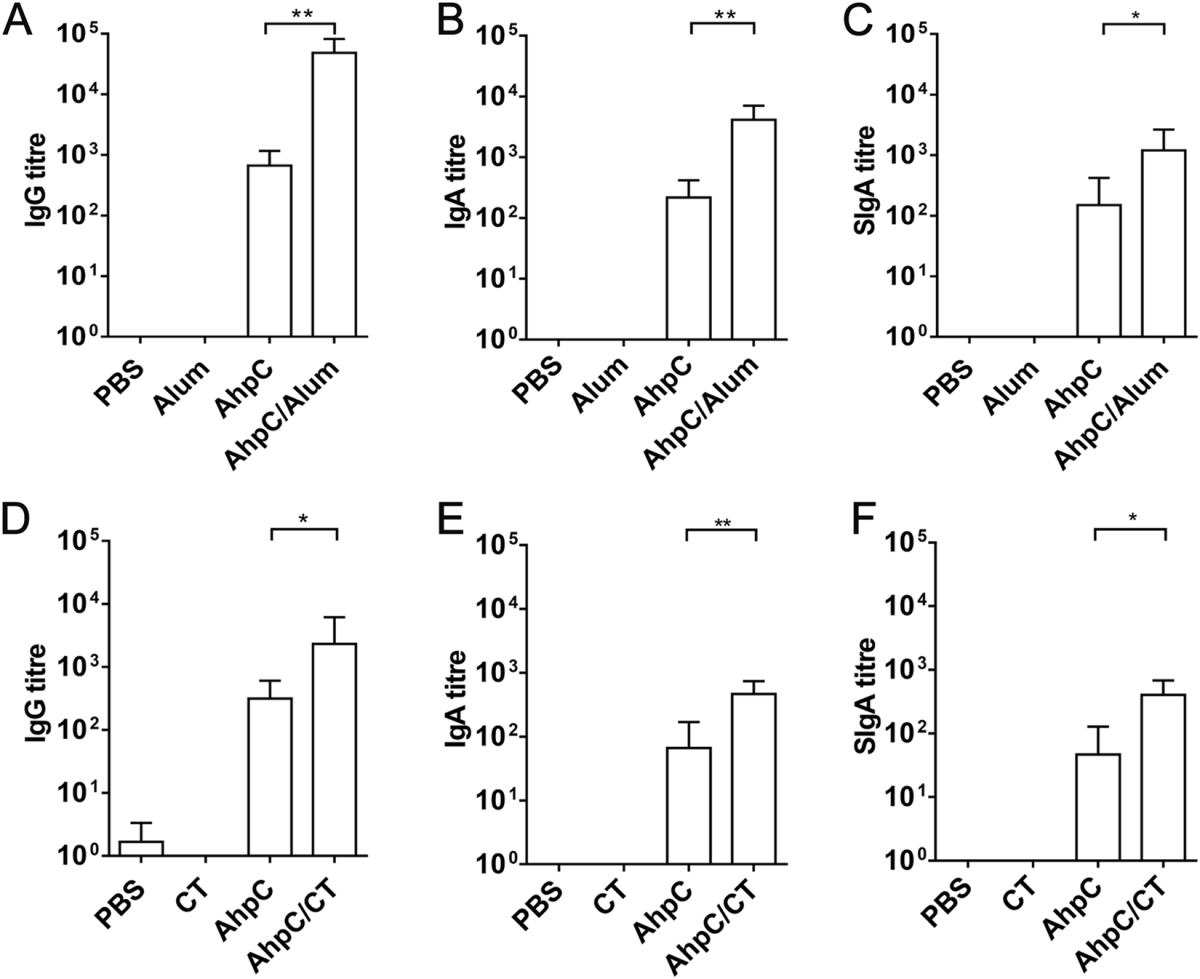
Quantitative antibody response to *F. nucleatum*-AhpC immunization. Mice were immunized by intraperitoneal injection (systemic immunization) (**A,B,C**) or by intragastric administration (mucosal immunization) (**D,E,F**) with PBS, adjuvant, Fn-AhpC or Fn-AhpC combined with alum or cholera toxin (CT). One week after final vaccination, anti-AhpC IgG and IgA titres in sera/intestinal mucus were determined using ELISA. *P < 0.05, **P < 0.01.

To investigate whether Fn-AhpC could induce strong systemic or mucosal responses, the mice were treated with recombinant Fn-AhpC (100 μg) with or without adjuvant/cholera toxin by intraperitoneal (*i.p*.) injection or intragastric (*i.g*.) administration, respectively (Table 1). Mice treated similarly with either adjuvant or PBS alone served as control groups. As shown in Fig. 3A–F, all mice immunized with Fn-AhpC elicited specific anti-Fn-AhpC antibodies, while mice solely immunized with PBS or adjuvant rarely showed any induction of an immune response. In addition, immunization with AhpC/alum by *i.p*. injection enhanced a stronger immune response to AhpC antigen and showed significantly higher serum IgG, IgA and intestinal mucus SIgA antibodies compared to those immunized with AhpC alone (Fig. 3A–C). Consistently, we detected higher titres of serum IgG, IgA and SIgA antibodies in mucosal immunization with AhpC/cholera toxin compared to immunization with AhpC alone (Fig. 3D–F). Notably, the titres of serum antibodies in the *i.p*. injection groups were markedly higher than those in the *i.g*. administration groups, indicating that mucosal immunization with Fn-AhpC elicited lower levels of antibodies compared with systemic immunization.

**Table 1:** Study time frames for the systemic and mucosal immunization studies.

	Route	Immunization (week)	Challenge (week)	Adjuvant	n
Systemic	Intraperitoneal injection (*i.p*.)	1, 3, 5	×3 doses (5)	Alum	10
Mucosal	Intragastric (*i.g*.)	1, 3, 5	×3 doses (5)	Cholera toxin	10

**Figure 4:**
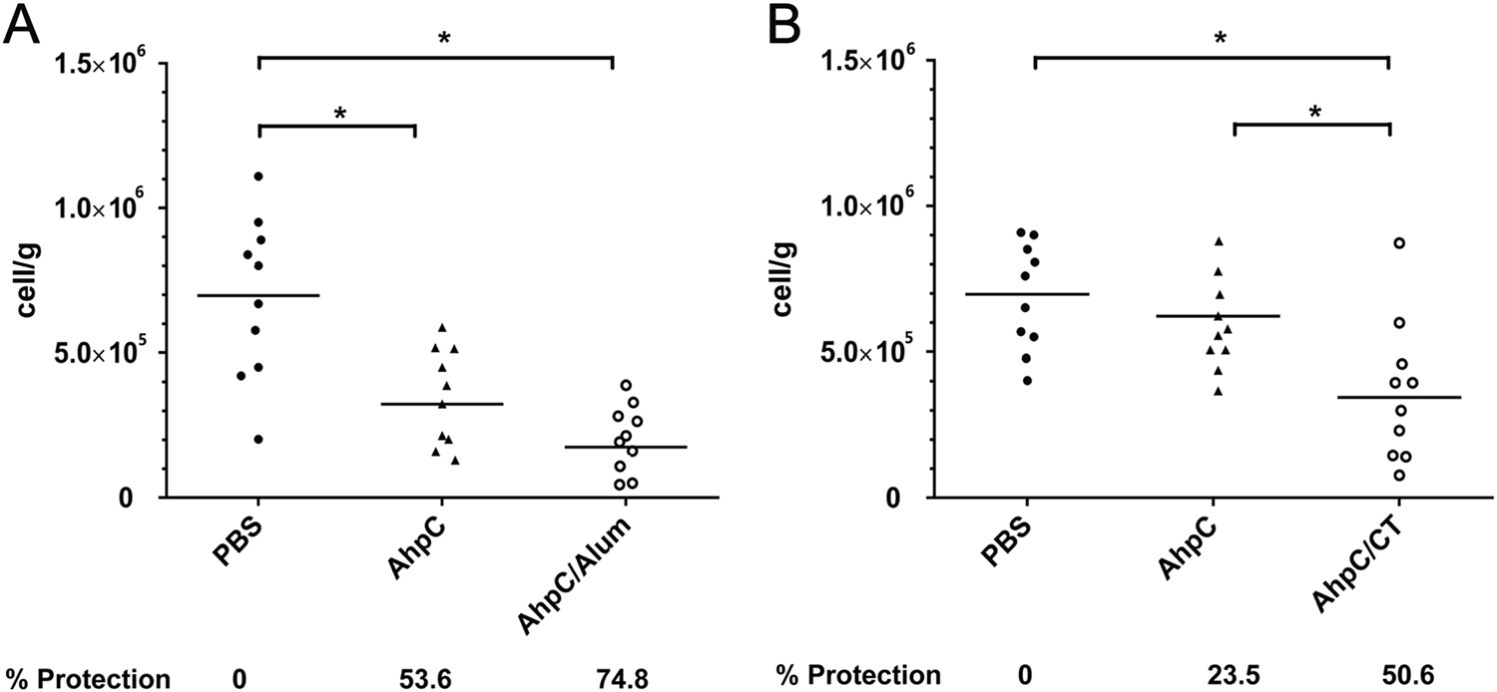
Induction of significant protection against *F. nucleatum* by immunization with *F. nucleatum*-AhpC vaccine. Mice were immunized by intraperitoneal injection (**A**) or intragastric administration (**B**) with PBS, AhpC or AhpC combined with alum or cholera toxin (CT). One week after the final vaccination, the mice were challenged with *F. nucleatum* and colonization quantified using qPCR assay. *P < 0.05, **P < 0.01.

Furthermore, the mice were challenged with live Fn by daily oral gavage, and the protective efficacy was determined according to the load levels of Fn within mice intestine tissues using qPCR. The results showed that 74.8% of mice immunized by *i.p*. injection with AhpC/alum obtained significant protection from infection with Fn, whereas only 53.6% of mice immunized with AhpC alone obtained protection against Fn infection (Fig. 4A). However, immunization by *i.g*. administration showed a lower efficacy in protecting from infection compared to immunization by *i.p*. injection. Approximately 50.6% of immunized animals were protected from infection after challenge with Fn in the AhpC/cholera toxin group, while only 23.5% of immunized animals were protected in the AhpC group (Fig. 4B). This finding indicated that *i.p*. injection elicited a more efficacious effect in inhibiting the intestinal colonization of Fn compared with the *i.g*. administration of AhpC/cholera toxin. These results demonstrated that systemic immunization with Fn-AhpC conferred stronger protection than mucosal immunization. The systemic immunization of Fn-AhpC/alum exhibited the most significant reduction in bacterial load, suggesting that the Fn-AhpC vaccine might have the potential to decrease Fn colonization in the digestive tract.

### Serological survey of antibodies against *F. nucleatum*-AhpC in CRC patients

**Figure 5:**
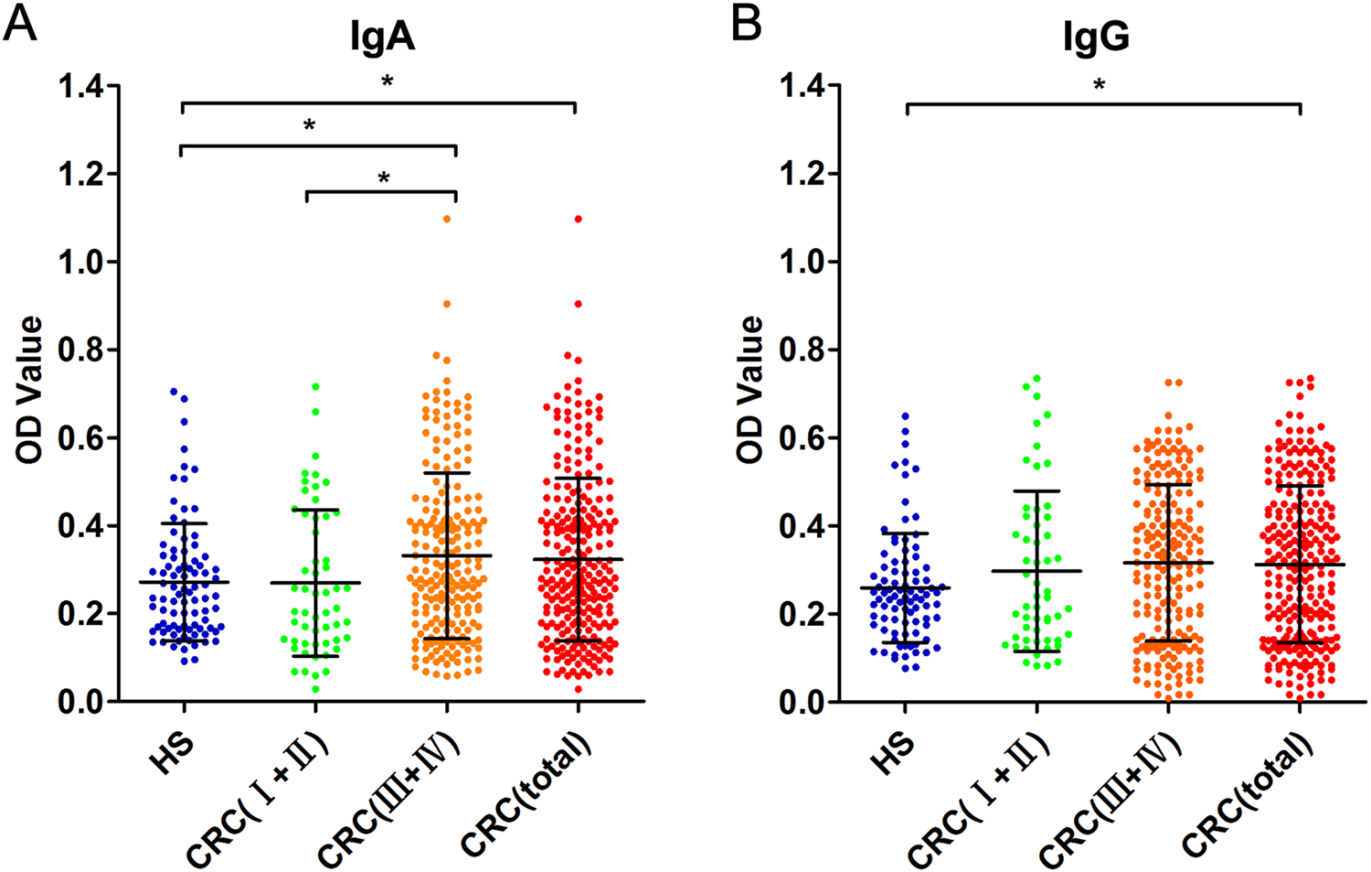
Comparison of serum levels of anti*-F. nucleatum*-AhpC from CRC patients and healthy subjects. Healthy subjects (HS, n = 92), stage I-II of CRC (n = 55), stage III-IV of CRC (n = 203), the total of CRC patients (n = 258) were individually assayed. Symbols indicate individual OD value; horizontal lines indicate the mean values ± SD. Differences between the four groups were analysed using the Kruskal-Wallis test. *P < 0.05. (**A**) anti-Fn-AhpC-IgA. (**B**) anti-Fn-AhpC-IgG.

To further analyse the anti-Fn-AhpC antibody levels in CRC patients, sera samples from CRC patients (n = 258) and healthy subjects (n = 92) were detected using indirect ELISA. The purified Fn-AhpC was coated as an antigen, while the diluted sera of patients and controls served as primary antibodies. The average absorbance for anti-Fn-AhpC IgA in the CRC group exhibited a significantly higher level than observed in healthy subjects (*P* = 0.018, *P* < 0.05) (Fig. 5A). The serum IgA levels of anti-Fn-AhpC in patients with late-stage CRC (stage III-IV, n = 203) were remarkably higher than those in healthy subjects (*P* = 0.007, *P* < 0.05) and in early-stage CRC patients (*P* = 0.026, *P* < 0.05). There was no significant difference between early-stage CRC patients (stage I-II, n = 55) and healthy subjects. However, a significantly higher level of anti-AhpC IgG was detected in all CRC groups compared to the healthy group (*P* = 0.001, *P* < 0.05). No significant difference was observed between early-stage and advanced CRC groups (Fig. 5B).

The associations between levels of anti-Fn-AhpC and clinicopathological parameters are presented in Supplementary Table 2. Neither anti-Fn-AhpC IgA nor IgG showed an obvious correlation with age, gender, tumour volume, histological differentiation, T classification, N classification, metastasis and tumour marker CEA or CA19–9. However, a significant association between the presence of anti-Fn-AhpC IgA and clinical stage (*P* = 0.026) was observed. Pearson’s correlation coefficient and linear regression analysis were applied to analyse the correlation between the levels of antibodies to Fn whole-cell and AhpC antigen. The levels of IgA antibody to AhpC were positively correlated with IgA antibody to Fn whole-cell (R = 0.149, *P* = 0.002), but the levels of anti-AhpC-IgG were not correlated with IgG to Fn whole-cell. (Supplementary Fig. 3). These results showed that the elevated antibody levels to Fn-AhpC only presented in late-stage CRC groups, suggesting that patients with early-stage CRC might lack the anti-Fn-AhpC antibody against Fn-AhpC.

### Antibodies of AhpC inhibited growth of *F. nucleatum in vitro*

**Figure 6:**
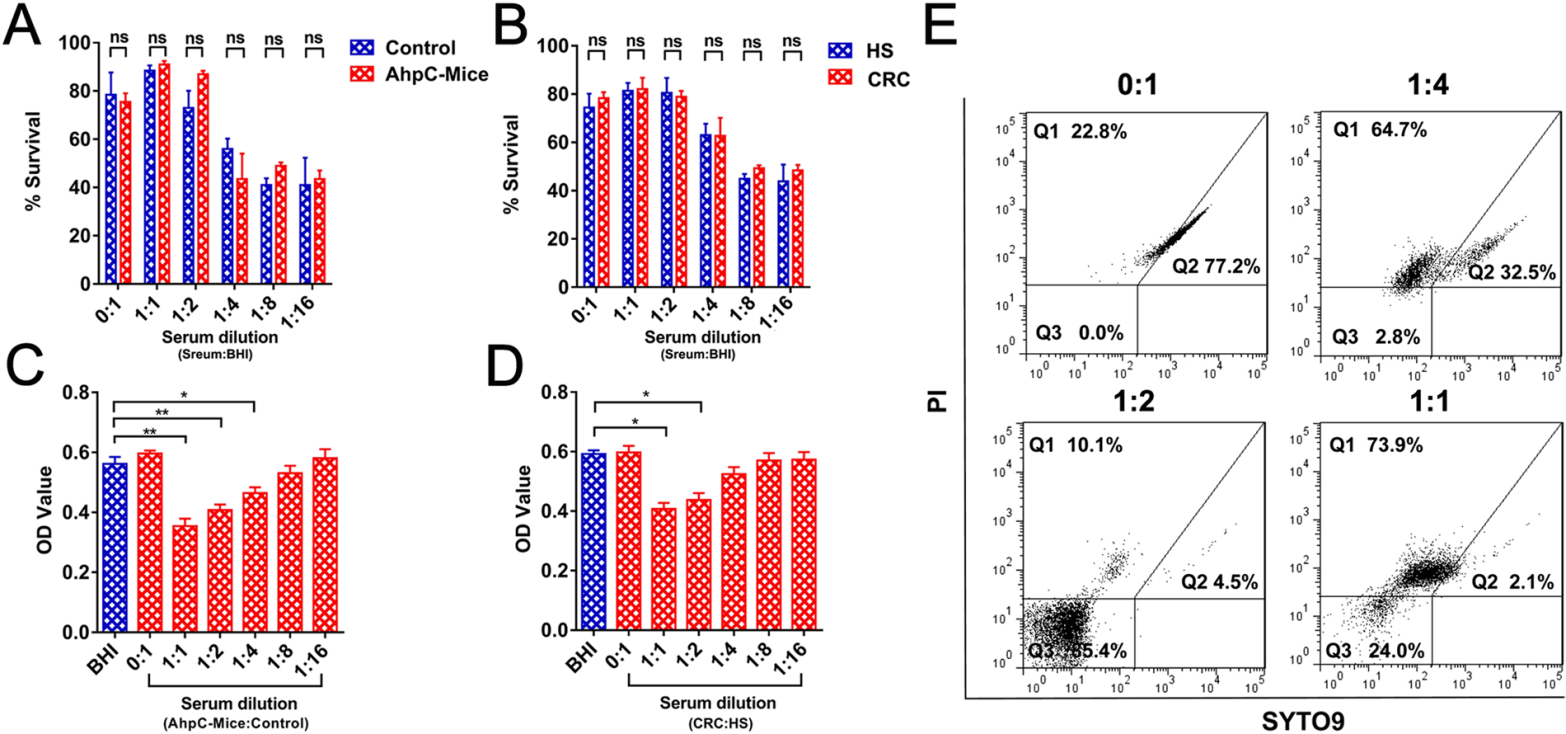
Serum with high titre antibodies to AhpC inhibited Fn growth *in vitro*. Survival of *F. nucleatum* strains was measured by bactericidal assays in serum of AhpC immunized and unimmunized mice (**A**) or CRC patients and healthy subjects (**B**). Inhibition of *F. nucleatum* strains was measured using antibacterial assays in the serum of AhpC immunized and unimmunized mice (**C**) or CRC patients and healthy subjects (**D**). Flow cytometric analysis of the stained Fn (**E**). Gates indicate the position and concentration of intact cells on the plots. Q1: dead cells, Q2: live cells; Q3: injured cells and debris. *P < 0.05, **P < 0.01, ns: no significant.

To investigate the bactericidal effect of anti-AhpC antibodies, Fn was incubated with pooled serum from mice immunized with AhpC at 37 °C for 30 min, and the serum from unimmunized mice was used as a control. The pooled serum was diluted 1:1 to 1:16, and there was no significant difference in survival between incubation with serum from immunized and unimmunized mice (Fig. 6A). However, media containing higher serum concentrations (>25%) improved the survival rate of Fn compared to media without serum. A similar result was observed for the Fn bacteria incubated with serum from healthy subjects compared to CRC patients with high titres of anti-AhpC antibodies (Fig. 6B).

Additionally, serum from immunized mice or CRC patients was two-fold serial diluted with serum from unimmunized mice or healthy individuals, respectively. Subsequently, Fn was cultured with 25% of the above pooled serum from mice for 48 h. The results showed that Fn cultured with immunized serum significantly inhibited Fn growth in a titre-dependent manner, while Fn cultured with serum from unimmunized mice did not show any growth inhibition (Fig. 6C). Furthermore, Fn was cultured in 25% pooled serum from CRC patients and healthy individuals for 48 h. These results showed that Fn growth was significantly inhibited when treated with sera from CRC patients with a high titre of anti-AhpC IgG and IgA, while Fn growth in sera from healthy subjects was not inhibited (Fig. 6D). The viability of Fn bacteria was further detected by flow cytometric analysis. The percentage of live Fn sharply decreased when treated with low anti-AhpC titre serum (1:4 dilution) (77% *vs*. 32.5%), and almost no bacteria survived (2.1%) when treated with high titre serum (1:1 dilution) (Fig. 6E). These results showed that antibodies to AhpC could inhibit Fn growth as an antibody-based AhpC inhibitor rather than playing a bactericidal role.

## Discussion

Emerging studies have demonstrated that Fn infection could drive strong immune responses^12, 13, 15, 23, 24^. Mice immunized with ultraviolet-inactivated Fn effectively minimized the progression of chronic halitosis associated with abscesses^25^. However, the whole-cell vaccine approach, including inactivated pathogens or live attenuated mutants, remains infeasible, reflecting the carcinogenicity of Fn. Thus, the development of safe subunit vaccines is a promising strategy to eliminate or eradicate Fn infection.

In the present study, we detected two candidate antigens, FomA and AhpC. Vaccination targeting FomA has previously been explored against bacterial biofilm formation and was found to be associated with pathogenicities, such as periodontal infection and halitosis^26, 27^. Oral administration of recombinant FomA-expressing *Lactobacillus acidophilus* was demonstrated to reduce the risk of periodontal infection^28^. Although FomA conferred a protective effect against bacterial co-aggregation with *Porphyromonas gingivalis*, recent studies have identified a mechanism involving the overabundance of Fn in tumourigenesis and metastasis^26, 27^. Thus, it is important to develop a new target as tumour vaccine to reduce the Fn load.

Oxidative stress is associated with malignant transformation^29^. In several intracellular bacterial pathogens, the antioxidant protein AhpC is unregulated as part of the response to host stress. *H. pylori*-AhpC has been demonstrated as a prognostic or diagnostic marker to monitor different stages of tissue damage from *H. pylori*-infected gastrointestinal diseases^30, 31^. In addition, AhpC is required for intestinal colonization and for the survival of *Helicobacter cinaedi* under oxidative stress^32^. Similarly, as an abundant and versatile antioxidant enzyme, Fn-AhpC is involved in protection against hydrogen peroxide damage and may be important for Fn survival. Theoretically, for obligate anaerobic bacteria, which are extremely sensitive to oxidative stress, the AhpC antibody could block the protective role against hydrogen peroxide damage, suggesting that AhpC may serve as a potential target for the development of vaccines against the survival of Fn.

Furthermore, as a potential vaccine against *H. pylori*-infection, *H. pylori*-AhpC stimulates strong adaptive immunity by decreasing the bacterial load in the stomach^22^. *Mycobacterium tuberculosis*-AhpC elicits a strong humoural immune response and stimulates T cells^33^. The AhpC protein was considered a virulence factor important for the survival of mycobacteria within macrophages. *S. typhimurium*-AhpC is induced during macrophage interactions, and both cell-mediated and humoural responses to AhpC were developed during the course of infection through T helper cell activation^34, 35^. Subsequently, we identified that Fn-AhpC was strongly immunogenic for mice model and seropositive human donors. We observed that Fn contained an AhpC homologue that showed extremely low sequence identity with human AhpC (31%) and moderate identity (50~70%) with the intestinal bacterium, although highly conserved homologues of AhpC are widespread throughout bacteria. The results of the sequence analysis suggested that antibody for Fn-AhpC could be specific, reflecting low cross-reactivity with the host or commensal bacteria. Consistent with this result, western blot assay showed that antibodies against AhpC were specific for the serum from CRC patients with Fn infections.

In general, an alternative approach to induce cellular immunity by subunit vaccination involves formulations containing adjuvants capable of promoting this type of response. IgG is the most prevalent serum antibody is used as the most common correlate of protection, and SIgA, secreted by the gut mucosa, is an important contributor to gut barrier function^36^. In the present study, systemic prophylactic immunization with AhpC/alum exhibited higher IgG levels and conferred protection against infection in 77.3% of mice, while mucosal immunization with AhpC/cholera toxin conferred partial protection against infection in 50.6% of mice and elicited low levels of serum IgG but higher SIgA compared with systemic immunization. These results showed that both serum IgG levels and intestinal mucus SIgA in AhpC vaccines could play important roles in reducing the Fn load.

Recent studies have reported that Fn-DNA is enriched in early-stage CRC^37^. Consistently, in a previous study, we showed an increase of the total antibody levels of Fn in early CRC patients^16^, indicating that although Fn induced both humoural and cellular immune responses in early-stage tumours, the host was incapable of eradicating the pathogen Fn. The serological survey in the present study also showed elevated anti-AhpC levels in only a few patients with late-stage of CRC, suggesting that Fn overload in CRC patients likely reflect a lack of protective antibodies, such as the anti-AhpC antibody.

Previous studies have shown that antibodies can inhibit bacterial and fungal infections directly and prior to attachment^38^. Mycograb is an antibody against fungal heat-shock protein 90 (HSP90), which directly inhibits the growth of *Candida in vitro* ^39^. HSP90 is essential for cell viability and can protect cells against oxidative stress. Interestingly, the extracellular Hsp90 proteins are secreted via a non-classical exosomal protein secretory pathway^40^. In addition, as an antibody fragment without Fc component, Mycograb plays an inhibitory role via the complement-independent pathway. The similar antioxidant function of HSP90 with AhpC implies a similar antibacterial mechanism by which anti-AhpC antibody may act as an antibody-based AhpC inhibitor.

In summary, we have shown that immunization with recombinant Fn-AhpC protein is able to induce strong humoural immunity and can suppress Fn loads in the mouse intestinal tract. The serological survey further showed a lack of anti-AhpC antibodies in early-stage CRC patients. Importantly, we found that serum with a high titre of anti-AhpC antibodies was able to inhibit Fn growth *in vitro*. These results demonstrated that AhpC constitutes a potential target for immune responses during Fn infection and represents a promising candidate for Fn vaccine. Future studies will address multivalent subunit vaccination using combinations of several virulence proteins to eradicate intracellular infections, thus enhancing the protective effects of AhpC vaccination.

## Materials and Methods

### Bacterial strain and culture conditions

Fn ATCC 25586 was purchased from the Institute of Microbiology of Chinese Academy of Sciences. The organisms were anaerobically grown at 37 °C for 48 h in brain heart infusion (BHI, Oxoid, Hampshire, UK) broth prior to harvesting.

### Patients, blood samples and ethical approval

Serum samples from 258 patients with primary CRC and from 92 healthy subjects were selected prior to surgery from an archive of blood samples at the Cancer Center of Sun Yat-sen University (SYSU), as previously described^16^. Ethics approval was granted by the Ethics Committee of SYSU Cancer Center (No. GZR2012–123). All experiments were performed in accordance with the approved guidelines and related regulations. Written informed consent was required for all patients involved in the present study.

### Phylogenetic analyses and antigenic determinants prediction of AhpC

Phylogenetic tree based on the sequence alignment of AhpC coding regions was constructed using the Neighbour-Joining (N-J) method in the MEGA 5.1 software^41^ with 1,000 bootstrap replicates. The hydrophilicity, surface probability and antigenic index plots of AhpC protein were predicted according to the methods of Jameson-Wolf, Emini and Hopp-Woods using the DNAStar protein analysis system Lasergene 14.1 (Madison, WI, USA). The dominant B cell epitopes of the AhpC proteins were selected through comprehensive analysis.

### Construction and purification of recombinant AhpC

The Fn-AhpC gene was PCR amplified using the following primer pair: 5′-CAG TAC GCA TAT GTC ATT AAT AGG AAG-3′ and: 5′-AGC GGA TCC TTA TAA TTC TCC AAT TAA ATC-3′, incorporating a *Nde*I and a *Bam*HI restriction site, respectively. The PCR products were double digested to generate sticky ends and ligated into a digested pET28a expression vector (Novagen, Inc., Madison, WI, USA). The vector construct was transformed into *E. coli* DH5α. The amplified pET28a construct was purified from the cultured cells and transformed into *E. coli* BL21. Subsequently, isopropyl-β-D-thiogalactoside (IPTG, 0.5 mM) was added to the culture for 16 h at 25 °C to induce the expression of AhpC, and the fusion protein from the supernatant of the IPTG-induced bacterium lysate was purified using a standard Ni-NTA agarose column purification protocol (Sangon Biotech, China).

### Recombinant AhpC identification by MALDI-TOF/TOF MS

The purified recombinant AhpC was extracted from gels stained with Coomassie brilliant blue R250 and digested with trypsin (Promega, USA) using the Mass Standards Kit for the 4700 Proteomics Analyser (Applied Biosystems); subsequently the tryptic protein hydrolysates were analysed using 4800 Plus MALDI TOF/TOF^TM^ Analyser (Applied Biosystems, USA). Protein identification was repeated at least twice using bands from two different gels. The peptide mass fingerprint (PMF) was obtained using Mascot 2.2 software to search Swiss-Prot and NCBI nr protein databases.

### Antibody detection using western blotting

Bacterial total protein or recombinant Fn-AhpC was separated using 10% SDS-PAGE and subsequently transferred onto PVDF membranes. After blocking with 5% non-fat dry milk in PBST, the membrane strips were incubated with pooled serum from 6 healthy subjects or separated serum from 6 Fn-positive CRC individuals as primary antibody (1:3000 dilution) at 4 °C overnight. Subsequently, the PVDF membrane strip was incubated with horseradish peroxidase (HRP) labelled anti-Mouse-IgA (1:8000) for 2 h. The bands were detected using Pierce ECL Plus Substrate (Thermo Scientific, USA) according to the manufacturer’s instructions.

### Animal used and ethical approval

Specific pathogen-free and age-matched 4- to 8-week-old female BALB/c mice were purchased from Sun Yat-Sen University (Guangzhou, China). All animal procedures were authorized by the Sun Yat-Sen University Animal Experimentation Ethics Committee and performed in accordance with the approved guidelines (Ethics number: IACUC- DD-16-0301).

### Immunization with AhpC and assessment of protective efficacy

Mice were immunized according to the schedule shown in Table 1. In the initial systemic immunization study, mice were immunized intraperitoneally with PBS, alum, AhpC (100 μg) or AhpC (100 μg) combined with alum (1:1, Imject Pierce, USA) at two weeks apart. In the mucosal study, the mice were immunized once a week with 3 orogastric doses containing PBS, cholera toxin (CT, 10 μg, Sigma-Aldrich), AhpC (100 μg) or AhpC (100 μg) combined with cholera toxin. At one week after the final immunization (week 6), the mice were challenged with Fn (1 × 10^8^ CFU/ml, 100 μl) by oral gavage daily for a week.

The load levels of Fn within mice intestines tissues were quantified by qPCR using SYBR Green PCR MasterMix (Applied Biosystems, USA). The following primers were used in the qPCR: Fn forward: 5′-ATA CCG GGA ATA AAG ACA-3′ and reverse: 5′-TAC AAC CCA ATC CAT AAG T-3′. Universal 16 S rDNA forward: 5′-CGC TAG TAA TCG TGG ATC AGA ATG-3′ and reverse: 5′-TGT GAC GGG CGG TGT GTA-3′^16, 42^. The copy numbers of Fn DNA and 16S rRNA were determined after serially diluting the standards (10^8^~10^1^ copies of plasmid DNA containing the respective amplicon). Subsequently, quantifications of Fn were normalized to the 16S rRNA copies as following: Fn copies = Fn_(target)_ copies × (10^8^/16S rRNA copies of total). Bacterial quantity was expressed as bacteria per gram of colon (approximately one copy per cell)^43^. The rate of protection was calculated using the following the formula: protection rate (%) = (infection percentage in the control group − infection percentage in the immunized group)/infection percentage in the control group × 100%^44^.

### Evaluation of serum antibody responses by ELISA

ELISA plates were coated with purified AhpC (0.5 μg/ml; 100 μl/well) overnight at 4 °C. Serum samples from CRC patients were diluted at 1:50. Sera extracted from immunized mice for 6 weeks were diluted from 1:100 to 1:12800. Intestinal mucus samples were collected from a 10 cm portion of the small intestine and diluted at 1:10 and at 1:4 serial dilutions. Subsequently, the samples were added in duplicates for 1 h at 37 °C, and the plates were washed three times with PBST. Next, 100 μl of goat anti-mouse IgG or IgA conjugated with HRP (1:8000, EarthOx, USA) was incubated at 37 °C for 30 min. Next, the plates were washed and the substrate (tetramethylbenzidine) solution was added, and subsequently, the reaction was terminated using 2 M H_2_SO_4_ and read at an OD of 450 nm using an ELISA spectrophotometer (Bio-Rad, USA).

### Serum bactericidal assays

Serum bactericidal assays were performed according to the method described by Lewis LA *et al*.^45^. Briefly, approximately 1 × 10^5^ CFU of Fn bacteria were incubated with the pooled serum (two-fold serial diluted in BHI) from immunized/unimmunized mice or serum from CRC patients/healthy subjects, respectively. After incubation at 37 °C for 30 min (t_30_) anaerobically, live bacteria with serial dilutions were counted using the standard plate count method. Survival ratio was calculated as the number of viable colonies at t_30_ relative to t_0_.

### Serum antibacterial assays

The pooled serum from immunized mice was serially diluted from 1:1 to 1:64 in serum from unimmunized mice. Similarly, the pooled serum from CRC patients was serially diluted from 1:1 to 1:64 in serum from healthy subjects. 1 × 10^5^ CFU of Fn bacteria were subsequently cultured with 25% pooled serum in BHI broth at 37 °C for 48 h anaerobically. The minimum inhibitory concentration (MIC) of the immunized mouse serum or CRC patient serum was determined according to the standard broth microdilution method. The lowest concentration that completely inhibited microbial growth, as determined by optical density measurements at 600 nm, was considered the MIC. All reactions were performed in triplicate. The viability of Fn bacteria was monitored using the LIVE/DEAD BacLight Bacterial Viability Kit (Thermo, USA) and detected using flow cytometry (Merck Darmstadt, Germany). All protocols were performed according to the manufacturer’s instructions. Viable bacteria were labelled with SYTO 9, whereas dead bacteria were labelled with propidium iodide (PI).

### Statistical analysis

All statistical analyses were performed using the SPSS 16.0 statistical software package (SPSS Inc., Chicago, IL). The relationships between the anti-AhpC antibodies and the clinicopathological features were analysed using the Mann-Whitney U test. Comparisons of Fn colonization numbers and the specific anti-AhpC antibodies for the different groups of immunized mice were assessed using a two-tailed Student’s t test. The results of the antibody and *F. nucleatum* load levels were expressed as the means ± standard deviation (SD). The correlation between the levels of antibodies to *F. nucleatum* bacteria and AhpC antigen in the serum of CRC patients was analysed using Pearson’s correlation coefficient and linear regression. The statistical limit for significance was *P* < 0.05.

## Electronic supplementary material

Supplementary File
