# Supplementary material for: Immunization with alkyl hydroperoxide reductase subunit C reduces Fusobacterium nucleatum load in the intestinal tract
Source: Sci Rep. 2017 Sep 5;7:10566. doi: 10.1038/s41598-017-11127-x (PMC5585165; doi:10.1038/s41598-017-11127-x)
Supplement: Supplementary file 1 — Supplementary File [file 41598_2017_11127_MOESM1_ESM.pdf]

Immunization with alkyl hydroperoxide reductase subunit C reduces *Fusobacterium nucleatum* load in the intestinal tract

Song-He Guo[1#], Hai-Fang Wang[1#], Zhi-Gang Nian[1], Yi-Dan Wang[3], Qiu-Yao Zeng[2], Ge Zhang[1*]

[1]Department of Microbial and Biochemical Pharmacy, School of Pharmaceutical Sciences, Sun Yat-sen University, Guangzhou, China

[2]Department of Clinical Laboratory Medicine, Sun Yat-sen University cancer center, Guangzhou, China, Guangzhou, China

[3]Department of School of Life Science, Sun Yat-sen University, Guangzhou, China

[#] Song-He Guo and Hai-Fang Wang contributed equally to this work.

[*] **Corresponding Author:** Zhang Ge, Department of Microbial and Biochemical Pharmacy, School of Pharmaceutical Sciences, Sun Yat-sen University, No.132 Waihuandong Road, University Town, Guangzhou 510006, China. Tel: 86-20-39943027; Fax: 86-20-39943021; E-mail: zhangge@mail.sysu.edu.cn

Supplementary Table 1. The prediction of antigenic determinants for the AhpC protein among *F. nucleatum* and other related bacteria.

n	***Fusobacterium nucleatum***	***Helicobacter pylori***	***Campylobacter jejuni***	***Streptococcus zooepidemicus***	***Bacillus anthracis***	***Burkholderia pseudomallei***
**1**	IGRKVPEFK	IAFDKRVKDFHEKGFN	PKDF	TNEDLKG	TEVKPFKA	SPIKPFKA
**2**	AFKKGEKDF	SIDTPVEEV	AFDKRYEEFKNRGI	TELGDLQEQYETLKSLG	QVTDESLKGKWS	QVSDETLKGKWS
**3**	TELEDLQDNYEAFKKEGAE	KNTPVEKGGIG	WKNTPVNEGG	AWHDDSDV	TELEDLQNQ	TELGDLAERYAE
**4**	WADHSERIKKVT	DITKSISRDYDVLF	LTKQIAR	QYVRKHPGEVCPAKWKEGAE	GDPTRTI	AWHDTSDTIA
**5**	VHDNGIGRAKELLRKLQG	DKGRNAEMLR	TNEHGEVC	LTPSL	VRNNPGEVCP	HPGEVCPAKWTPGADTLTPSL
**6**	FVAEHGEVCPAKWQPGSETLKPSL	GWRKGDKGMKA	AGWNKGDEGMKANPKG		KWQEGSATLKPSL	
**7**			YLGKNEAKL			

n	***Salmonella typhimurium***	***Lactobacillus brevis***	***Escherichia coli***	***Bifidobacterium longum***	***Bacteroides fragilis***	***Clostridium butyricum***
**1**	KIKPFKNQAFKNGEF	TELGDLADH	TKIKPFKNQAFKNGEF	ELTDFK	QMPEFK	INKKVSDF
**2**	EVTEKDTEGRW	QADTEI	EITEKDTEGRW	QNNEFHEVTKDDV	QNGSFKTVSSEDVKG	YQNGEFKEVTLESIKG
**3**	GIGRDASDLLRKIKA	SISEDTEF	TELGDVADHYEELQKLG	TELEDLAENYAKFKEIG	ELVDVAEKYEQ	ADPTGRLARDFEVMIEEEGLAL
**4**	DPTGALTRNFDNMREDEGLADRA	EQSAEVGK	AWHSSSETIA	WHDANEKIAK	NPEGKIKIAEIQDNNIGRNA DELLRKVEA	HDLGIGRSADELLRKVQ
**5**	HPGEVCPAKWKEGEA	DVLN	DPTGALTRNFDNMREDEGLA DRA	KDLDTYNEADGVAERGDFIVSP EGKVV	KWKKGEATLKPSI	VAEHGDQVCPAKWQPGE
**6**		DPQGTVRS	HPGEVCPAKWKEGEA	SSNVGRNAEELLRRVQ		
**7**		GEQTLKPGTDLVG		TPGEETIEPSL		

Supplementary Table 2. Relationship between the OD value of IgG/IgA antibodies against Fn-AhpC and the clinicopathological variables in 258 patients with CRC.

Characteristics	n	IgG (Means±SD)	P	IgA (Means±SD)	P
Gender			0.643		0.471
Male	144	0.313±0.298		0.307±0.195	
Female	114	0.285±0.298		0.324±0.186	
Age (y)			0.288		0.068
<60	153	0.323±0.295		0.297±0.186	
≥60	105	0.255±0.241		0.341±0.196	
Tumour volume (cm^3)			0.163		0.324
≥8.0	83	0.247±0.230		0.338±0.205	
<8.0	175	0.339±0.304		0.311±0.189	
Histological differentiation			0.587		0.424
Well	25	0.338±0.314		0.322±0.187	
Moderate or poor	233	0.258±0.355		0.350±0.181	
pT classification			0.315		0.556
T1+T2	65	0.234±0.269		0.299±0.199	
T3+T4	193	0.309±0.272		0.316±0.186	
pN classification			0.130		0.285
No	152	0.326±0.310		0.299±0.168	
Yes	106	0.235±0.190		0.324±0.193	
pM classification			0.108		0.147
No	205	0.323±0.285		0.308±0.186	
Yes	53	0.205±0.229		0.368±0.199	
Stage			0.125		0.026
Ⅰ+Ⅱ	55	0.335±0.303		0.282±0.153	
Ⅲ +Ⅳ	203	0.245±0.219		0.328±0.196	
CEA (μg/ml)			0.750		0.352
< 5	146	0.310±0.291		0.295±0.201	
≥ 5	112	0.290±0.267		0.318±0.175	
CA19-9 (U/ml)			0.761		0.068
< 35	206	0.297±0.279		0.293±0.188	
≥ 35	52	0.319±0.281		0.350±0.197	

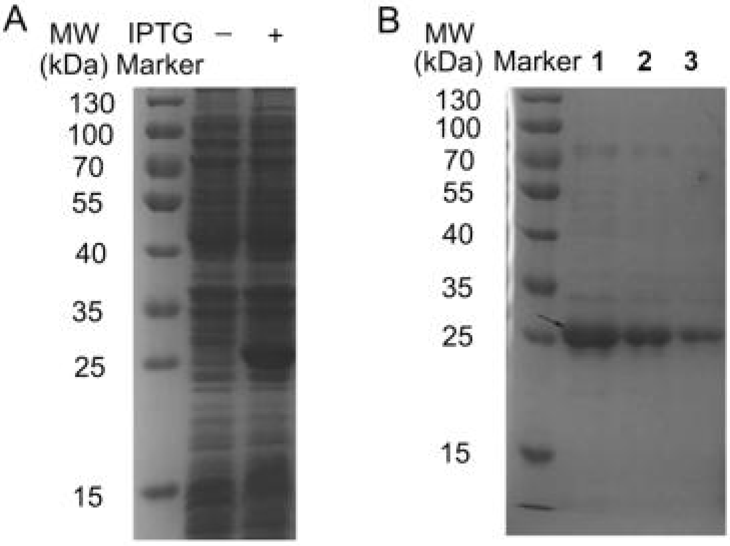

Supplementary Figure 1. Complete figures showing the expression of recombinant Fn-AhpC after IPTG induction are provided in Fig. 1A and Fig. 1B. (A) Recombinant AhpC expression in the absence (−) or presence (+) of 0.5 mM IPTG was detected using 12% SDS-PAGE and Coomassie brilliant blue staining. (B) The purification of recombinant AhpC. The recombinant AhpC with a His tag was purified using a Ni-NTA column after IPTG induction. Supplementary Fig. 1A was included in Fig. 1A of this manuscript; lane 2 in Supplementary Fig. 1B was included in Fig. 1B of this manuscript.

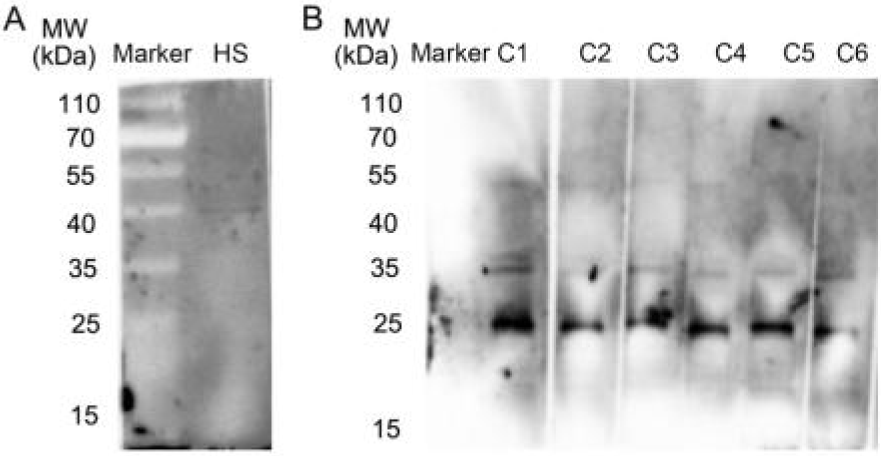

Supplementary Figure 2. Complete figures of the antigens reactive with anti-AhpC-IgA in Fig. 1D are shown. (A) The membrane strips were incubated with pooled serum from 6 Fn-negative healthy individuals. (B) The membrane strips were incubated with separated serum from 6 Fn-positive CRC patients (lane C1-C6). Notably, the gels in Supplementary Fig. 2 were run under the same conditions, and Western blotting was performed using the same set of materials. Supplementary Fig. 2 was included in Fig. 1D of this manuscript.

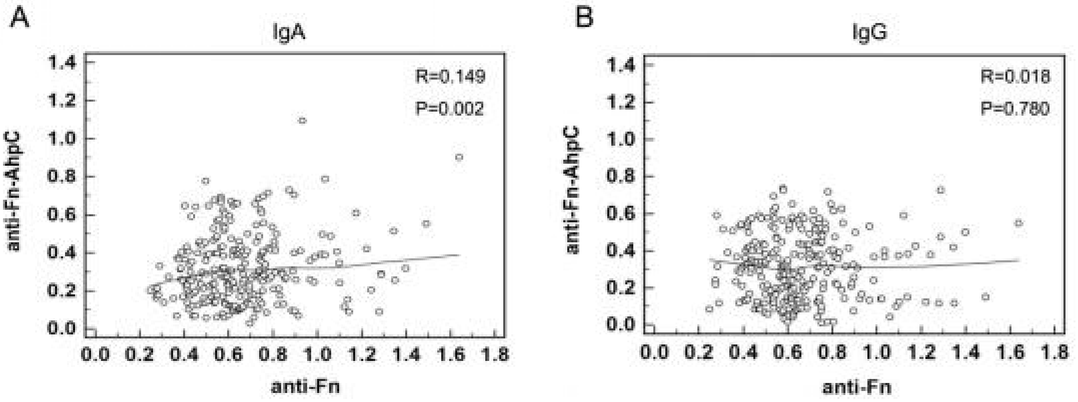

Supplementary Figure 3. The correlation between the levels of antibodies to *F. nucleatum* bacteria and AhpC antigen in serum of CRC patients were analysed using Pearson's correlation coefficient and linear regression.
